# Multiplexed clonality verification of cell lines for protein biologic production

**DOI:** 10.1002/btpr.2978

**Published:** 2020-02-17

**Authors:** Sofie A. O’Brien, Juhi Ojha, Paul Wu, Wei-Shou Hu

**Affiliations:** 1 Department of Chemical Engineering and Materials Science, University of Minnesota, Minneapolis, MN 55455-0132, USA; 2 Bayer HealthCare, 800 Dwight Way, Berkeley, CA 94710

**Keywords:** CHO cells, Cell culture, Clonality, Next-generation sequencing, Genomics

## Abstract

During the development of cell lines for therapeutic protein production, a vector harboring a product transgene is integrated into the genome. To ensure production stability and consistent product quality, single-cell cloning is then performed. Since cells derived from the same parental clone have the same transgene integration locus, the identity of the integration site can also be used to verify the clonality of a production cell line. In this study, we present a high-throughput pipeline for clonality verification through integration site analysis. Sequence capture of genomic fragments that contain both vector and host cell genome sequences was used followed by next-generation sequencing to sequence the relevant vector-genome junctions. A Python algorithm was then developed for integration site identification and validated using a cell line with known integration sites. Using this system, we identified the integration sites of the host vector for 31 clonal cell lines from five independent vector integration events while using one set of probes against common features of the host vector for transgene integration. Cell lines from the same lineage had common integration sites, and they were distinct from unrelated cell lines. The integration sites obtained for each clone as part of the analysis may also be used for clone selection, as the sites can have a profound effect on the transgene’s transcript level and the stability of the resulting cell line. This method thus provides a rapid system for integration site identification and clonality verification.

## INTRODUCTION

Chinese hamster ovary (CHO) cells are one of the most commonly used cell lines used to produce therapeutic proteins.^1^ They acquire the capability to produce these proteins by the introduction of a vector carrying the product gene and integration into the genome. After transfection, the resulting cells are heterogeneous and have a wide range of productivity and other properties. Single cell cloning is performed, and high producing cell lines are isolated as candidates to be the production cell line. To ensure the consistency and quality of the products produced by the cells over time, regulatory agencies require the demonstration of clonality, i.e. that cells originated from a single transfected ancestor cell.^2^

After transfection and entry into the nucleus, the vector integrates into the genome of the host cell randomly. One cell may have one or more integration events depending on the vector used and its dose. The chance of two integration events occurring on the same site, either on both alleles of the same chromosome or in the genome of two different cells, is extremely low. A characteristic of clonally derived cells is thus that all cells within the population should have the same integration site(s) of the vector on the genome. Identifying the integration site of the product gene and demonstrating that two sublines of cells have the same integration site can thus be taken as evidence that the two originated from the same ancestor. Furthermore, the genome context of the integration site can be explored to reveal information on epigenetic accessibility, transcriptional activity,^3^ and even stability of the region.^4^

Several PCR-based assays such as inverse PCR,^5^ splinkerette-PCR,^6^ linear amplification mediated PCR (LAM-PCR),^7^ and targeted locus amplification (TLA)^8^ have been developed to identify integration sites of transgenes. These methods can be applied to demonstrate clonality. LAM-PCR for integration site identification has been used to analyze and track clonal lineages of blood cells following lentiviral gene therapy,^9,10^ while TLA has been used in CHO cells for identification of clones with the same integration sites after pool selection and single cell cloning.^11^

In this study, we established a high throughput method that can be used to support monoclonality in different production cell lines generated using random integration of plasmids. By designing one set of probes for the common features of the host vector used to introduce the gene of interest, selective capture was utilized to simultaneously sequence vector-containing DNA from dozens of clonal cell lines from multiple independent vector integration events with different product genes. This method also allowed for a relatively simple bioinformatic analysis to identify these integration sites through the use of a Python algorithm. Cell lines were separable based on their integration sites, and we were able to demonstrate the clonality of the original cell populations. This method thus provides a rapid and cost-efficient tool for clonality verification and integration site identification in product producing CHO cells.

## MATERIALS AND METHODS

### Cell Line Construction

**Figure 1.**
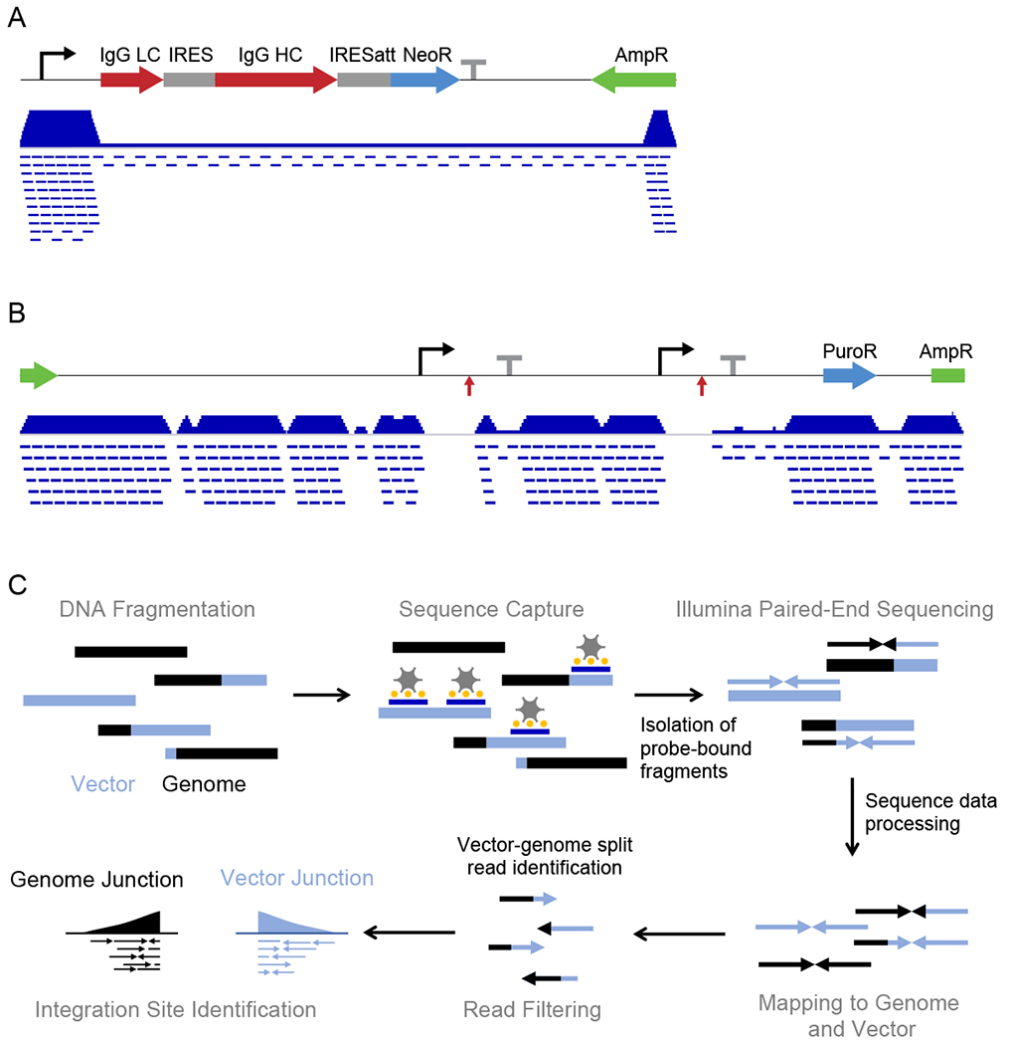
Overview of sequence capture method used. Probe coverage for SH-87 vector **(A)** and host vector from clonality analysis **(B)**. The vector maps are shown at the top, with key features illustrated. The probe coverage at each location of the vector is shown below the vector map as a pileup ranging from zero to nine **(A)** or zero to five **(B)**, and individual probe locations are denoted by short blue dashed lines below the pileup. Vector description for **(A):** A CMV promoter (black solid angled arrow) drives expression of Immunoglobulin G Light Chain (IgG LC, red arrow), Immunoglobulin G Heavy Chain (IgG HC, red arrow), and Neomycin resistance (NeoR, blue arrow), all linked by IRES elements (grey boxes), and followed by a SV40 early polyadenylation signal (grey solid t-shaped bar). The bacterial resistance marker (Ampicillin resistance, green arrow) is at the end of the vector next to the linearization site. Vector description for **(B):** The vector is linearized at the bacterial resistance marker (green arrow) and contains two sets of identical promoters (black solid angled arrows) and SV40 late polyadenylation signal terminators (grey solid t-shaped bars), as well as puromycin-N-acetyltransferase for selection (blue arrow). The product gene(s) are inserted between the promoters and terminators (red arrows). **(C)** Overview of experimental and bioinformatic methods used for integration site identification.

The monoclonal antibody producing cell line, SH-87, has been described previously.^12,13^ The organization of the tricistronic vector used for introducing the transgene is illustrated in Figure 1A.

Cell lines used for clonality analysis were all derived from the same CHO-K1 host cell line using the vector shown in Figure 1B with product gene(s) inserted between the illustrated promoters and terminators. After transfection with linearized plasmid by electroporation, cells were selected using Puromycin in 96 wells, and surviving clones were isolated after monitoring by a CloneSelect Imager (Molecular Devices, San Jose, CA). Subsequent subcloning was performed via limiting dilution.

### Probe Design

For integration site analysis of cell line SH-87, probes (120bp in length) were focused on the ends of the linearized vector (Figure 1A). The first 900bp and the last 400bp of the linearized vector had 9x coverage with tiling, with the remainder of the vector having 1x coverage.

For clonality analysis of platform cell lines, probes (120bp in length) were designed to capture the regions of the linearized vector that are common in all transfected cell lines at 5x coverage with tiling (Figure 1B). Sequences specific to individual cell lines, including that of the product gene, were thus not included in the probe design. Features in the vector with multiple occurrences, including the promoter and terminator, had reduced probe coverage.

### Experimental methods

An overview of the experimental methods is shown in Figure 1C. Genomic DNA (gDNA) from SH-87 was provided courtesy of Dr. Yuansheng Yang and Dr. Dong-Yup Lee from ASTAR, Bioprocessing Technology Institute, Singapore. gDNA was sheared to an average length of 500bp, and library preparation was performed using an Agilent SureSelectXT Reagent kit (Agilent # G9611A, Santa Clara, CA). DNA fragments containing vector sequence were captured using the Agilent SureSelect Enrichment system. Briefly, short biotinylated RNA probes were hybridized to the pooled gDNA library, and streptavidin conjugated beads were used to capture DNA fragments that had hybridized to the probes. Captured DNA was eluted, amplified, and sequenced on half a lane of Illumina MiSeq (Illumina, San Diego, CA) using 250bp paired end reads for a total of 12.7 million reads.

For all other cell lines, gDNA was extracted using a Qiagen Blood and Cell Culture DNA Max Kit (Qiagen #13362, Valencia, CA). 100ng of gDNA from each cell line was sheared using a Covaris E220 ultrasonicator (Covaris, Woburn, MA) to obtain fragments with an average length of 250bp. Further library preparation was performed using an Agilent SureSelect HS Reagent Kit (Agilent #G9702A, Santa Clara, CA). Libraries for all cell lines were pooled prior to sequence capture following the Agilent SureSelect system instructions. Captured DNA was eluted, amplified, and sequenced on one lane of Illumina MiSeq (Illumina, San Diego, CA) using 100bp paired end reads for a total of 25 million reads.

### Data Pre-processing

Fastq files from sequencing were trimmed to remove adapter sequences using Trimmomatic^14^ (version 0.33). Reads were then mapped to the CriGri-PICR release of the Chinese hamster genome^15^ using BWA-MEM^16^ (BWA release 0.7.17). The host vector sequence was added to the genome sequence as an additional scaffold. Duplicate reads were removed using the MarkDuplicates command in Picard tools 2.18.16. The resulting SAM (Sequence Alignment/Map) file, which describes how reads are aligned to the genome, was used for further analysis. Samtools^17^ (version 1.9) was used to create BAM files for visualization in IGV^18^ (version 2.4.19).

## RESULTS AND DISCUSSION

### Development of an integration site analysis pipeline

**Figure 2.**
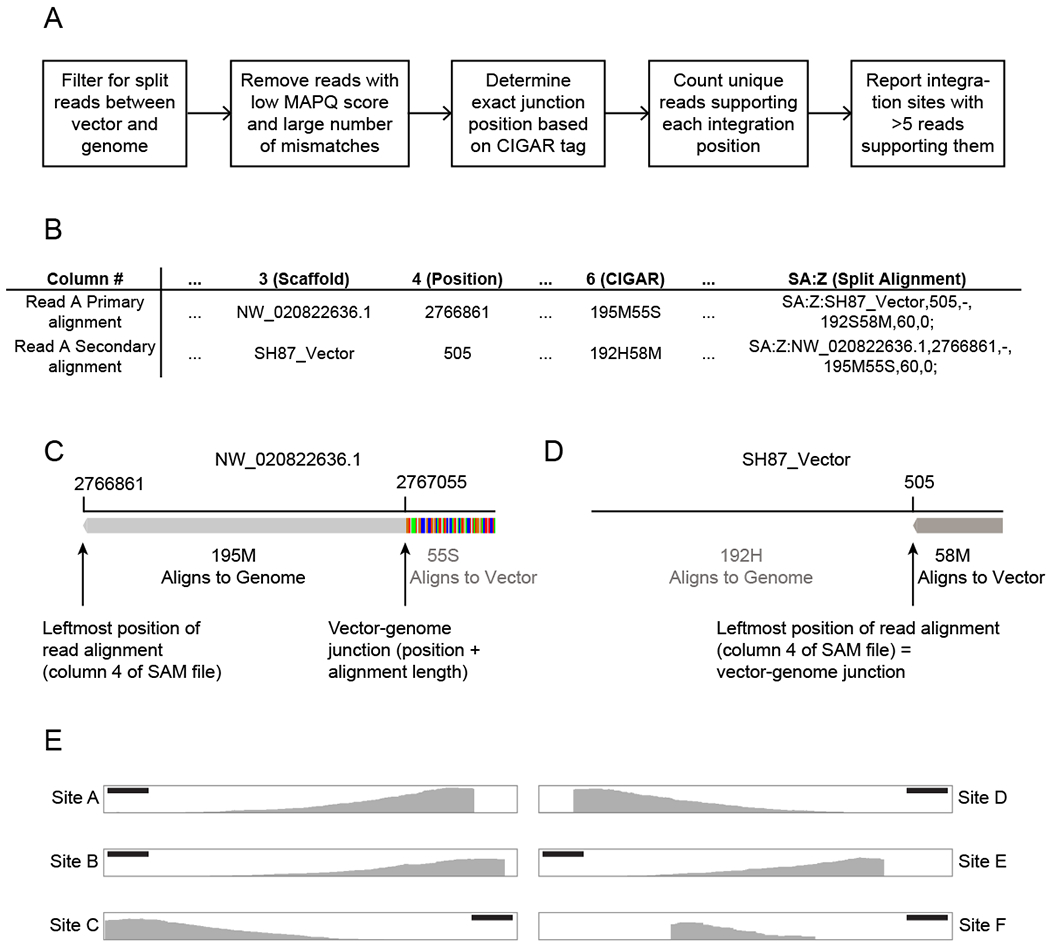
Integration site analysis pipeline and validation. **(A)** Procedure to identify integration sites. **(B)** SAM file alignments for a split read from mapped SH-87 sequence capture data. Primary and secondary alignments for the read are shown, along with select columns. **(C)** Illustration of primary alignment from (B) to Chinese Hamster genome. Vector-genome junction position is adjusted to account for portion of read aligned to genome. **(D)** Illustration of secondary alignment from (B) to SH-87 vector. Vector-genome junction does not need to be adjusted and is equal to the position listed in the SAM file. **(E)** IGV read pileups of identified integration sites for SH-87. Scale bar represents 50bp.

An analysis pipeline was written in Python 3.6.3 to identify integration sites from mapped sequencing data (Figure 2A, algorithm is available at https://doi.org/10.13020/9wgm-mj51). Sequence capture data from cell line SH-87 was used for validation of the method, as integration sites from this cell line had been previously determined using whole genome sequencing and confirmed by PCR/Sanger sequencing.^13^

The algorithm initially utilizes columns 3 and 4 (Scaffold and position) of the SAM file and the SA:Z tag added by BWA-MEM for chimeric alignments to identify reads which contain a split alignment between the vector and genome (Figure 2B). These alignments were then filtered for MAPQ (mapping quality score) > 30 and NM (number of mismatches) < 4. Reads mapping only to the genome, only to the vector, and unmapped reads were identified and counted for determining the number of on-target reads from sequence capture. For SH-87, there were 2.39 x 10^6^ unique read pairs, and 18.4% of the read pairs mapped to the vector (see Table S1 for detailed mapping statistics). The low percentage of on-target reads was compensated by the high sequencing depth, providing a sufficient number of split-reads for integration site analysis.

Next, the CIGAR (Concise Idiosyncratic Gapped Alignment Report) tag for each alignment was used to find the exact vector-genome junction position. The CIGAR string is a compact method to report how bases within a read align to the reference genome, specifying which bases match, are deleted/inserted, or are clipped (not aligned) in the case of split reads. Column 4 of the SAM file format reports the leftmost (smallest number) position that is aligned for the read alignment. If the CIGAR string begins with an alignment match (M), the length of the match is added to the position to obtain the vector-genome junction (Figure 2C). Otherwise, if the CIGAR string begins with bases that are not aligned (hard (H) or soft (S) clipping), the reported position is the first base that aligns, and thus is the location of the vector-genome junction (Figure 2D). Using this method, the position of the junction on the vector and genome for each read is identified, and the number of reads supporting each unique junction is tabulated. For paired end reads, if both ends of the pair support the junction, they were considered as only one count.

Due to the high sequencing depth of SH-87, a minimum of 15 reads or pairs of support were required for a vector-genome junction to be called an integration site. Previously, six vector-genome junctions from integration of the tricistronic vector in cell line SH-87 were identified by whole genome sequencing.^13^ All six junctions were found by the algorithm (Table S2). To confirm these results, the genomic integration sites were visualized using IGV (Figure 2E). Each integration site has a sharp boundary at the vector-genome junction. Additionally, the read pileup depth decreases as the distance from the integration site increases, as would be expected since the probability of a read being captured by vector probes diminishes with increasing distance from the junction. By using sequence capture and the Python algorithm, we were successfully able to determine the integration sites for SH-87 at a much lower sequencing and bioinformatic cost than whole genome sequencing.

### Application of pipeline for confirmation of clonality

To apply this method to the confirmation of clonality, gDNA of 31 different cell lines derived from five independent clonal cell lines (denoted as cell lineages A-E) were used in this study. Cell lines characterized from lineages A, B, and E consisted of a clonal parental cell line and a set of subclones. Unrelated cell lines C-1 and D-1 were added to the analysis to increase the diversity of cell lines examined. Cell line A-1 was run in duplicate (labeled A-1 and A-1D) for a total of 32 samples. gDNA from these samples was sheared, and fragments containing probed vector regions were captured and sequenced.

After sequencing, the library size (number of unique read pairs) for each sample ranged from 0. 46 - 5.6 x 10^5^ (Figure S1A). The percent of on-target reads (reads which mapped either entirely or partially to the vector) was greater than 50% for all samples, with the majority of samples having >70% of their reads containing vector sequence (Figure S1B). This corresponded to an enrichment ratio (reads mapped to vector/reads not mapped to vector) of between 1.2 and 18x, depending on the sample.

**Figure 3.**
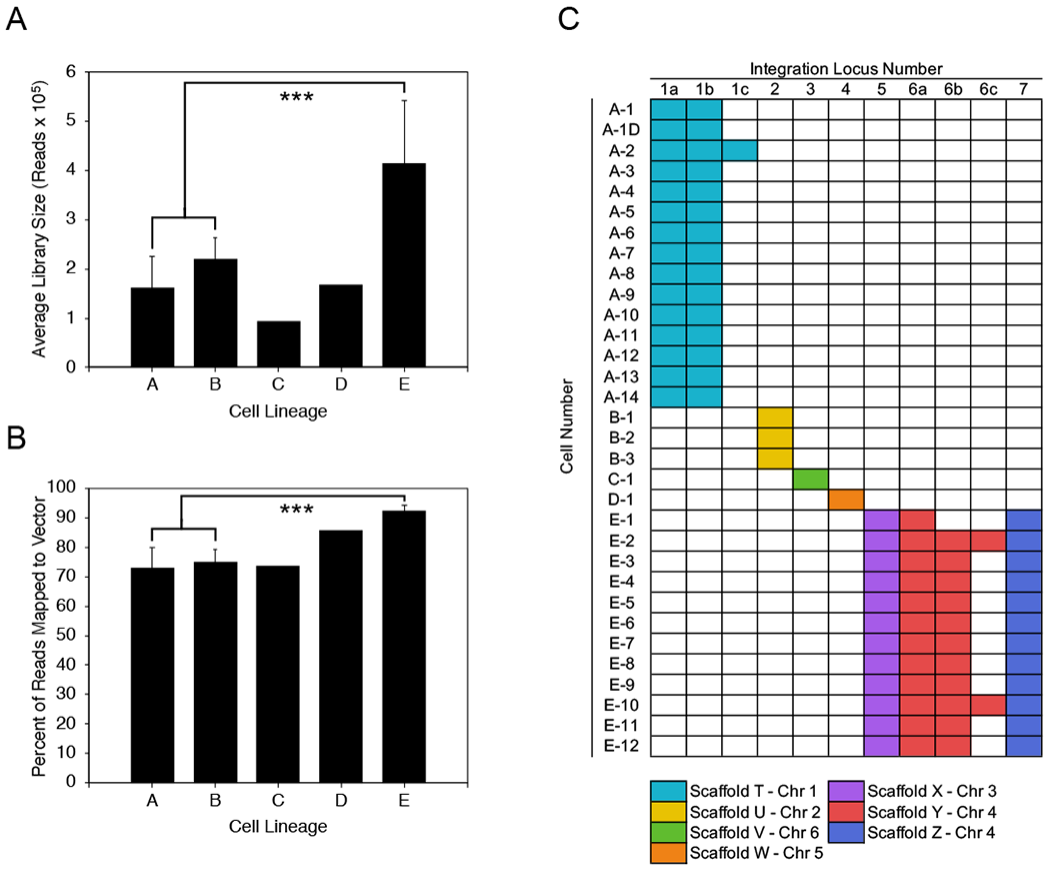
**(A)** Average number of unique reads for cell lines from each cell lineage (Number of cell lines for each lineage: A, n=14; B, n=3; C, n=1; D, n=1; E, n=14). *** *p* < 0.002, t-test. **(B)** Average percent of reads mapped to the vector for different cell lineages (*** *p* < 0.002, t-test). **(C)** Result of integration site identification. Each unique vector-genome junction is represented as an integration locus (numbered 1-7), and integration loci are colored by scaffold. The scaffolds and corresponding chromosomes are labeled below the chart.

The library size varied with cell lineage, with the cell lines from lineage E having on average significantly more unique reads after sequencing than those from lineage A or B (Figure 3A, t-test, p<0.002). Only one cell line was analyzed from each of cell lineages C and D, so these were not included in the comparison. The percent of on-target reads mapping to the vector also varied. Samples of cell lines from lineage E had a significantly higher percent of vector reads on average than lineages A or B (Figure 3B, t-test, p<0.002). There are several potential reasons for the bias in on-target read percentage and library size. gDNA from all cell lines was pooled at equal amounts after barcoding, so it is likely that inherent differences in the genome accounted for this difference. Cell lines from lineage E had three integration loci as opposed to the one locus found in populations from lineages A-D, and thus gDNA samples from lineage E would have higher vector sequence content. Despite this variability, the sequencing depth for each sample was sufficient for integration site analysis.

### Cell lines from the same lineage have common integration sites

The results of the integration site analysis are shown in Figure 3C and Table S3. As the library size for these cell lines was lower, a minimum of five reads or pairs of support were required for a vector-genome junction to be called an integration site. Each integration site identified was on a different scaffold. Cells from lineage A had one integration locus, and both ends of the vector insertion were identified, approximately 30bp apart. One end of a single integration locus was called by the algorithm for cells from lineages B, C, and D. This may be a result of complex sequence rearrangements, such as concatenations of the fragmented vector, at the other end of the vector-genome junction that would prevent proper mapping of the integration site. Incomplete mapping would also occur if the vector-genome junction was in a location that either was not probed or was not included in the vector sequence for mapping (such as the product gene), preventing the capture or mapping of DNA fragments from the integration site region. Three integration loci on different scaffolds were called for cells from lineage E, with both ends of the insertion found approximately 100bp apart for one of the three loci. Importantly, cell lines from the same lineage were found to have the same set of integration sites.

Several cell sublines (A-2, E-2, and E-10), had an additional vector-genome junction on the same scaffold as the integration site, either 1kbp (Site 1C for A-2, Figure 3C) or 4.7kbp (Site 6C for E-2 and E-10, Figure 3C) downstream of the main integration locus. As these additional vector-genome junctions were very close to the prevalent integration locus, it is highly unlikely that these are independent integration events. Rather, this could be the result of a genomic rearrangement or duplication in that scaffold that resulted in an additional integration junction, even though amplification was not performed during cell line development. Genomic heterogeneity has been previously shown after subcloning, with different subclones presenting a gain or loss of transgene copies.^4^

**Figure 4.**
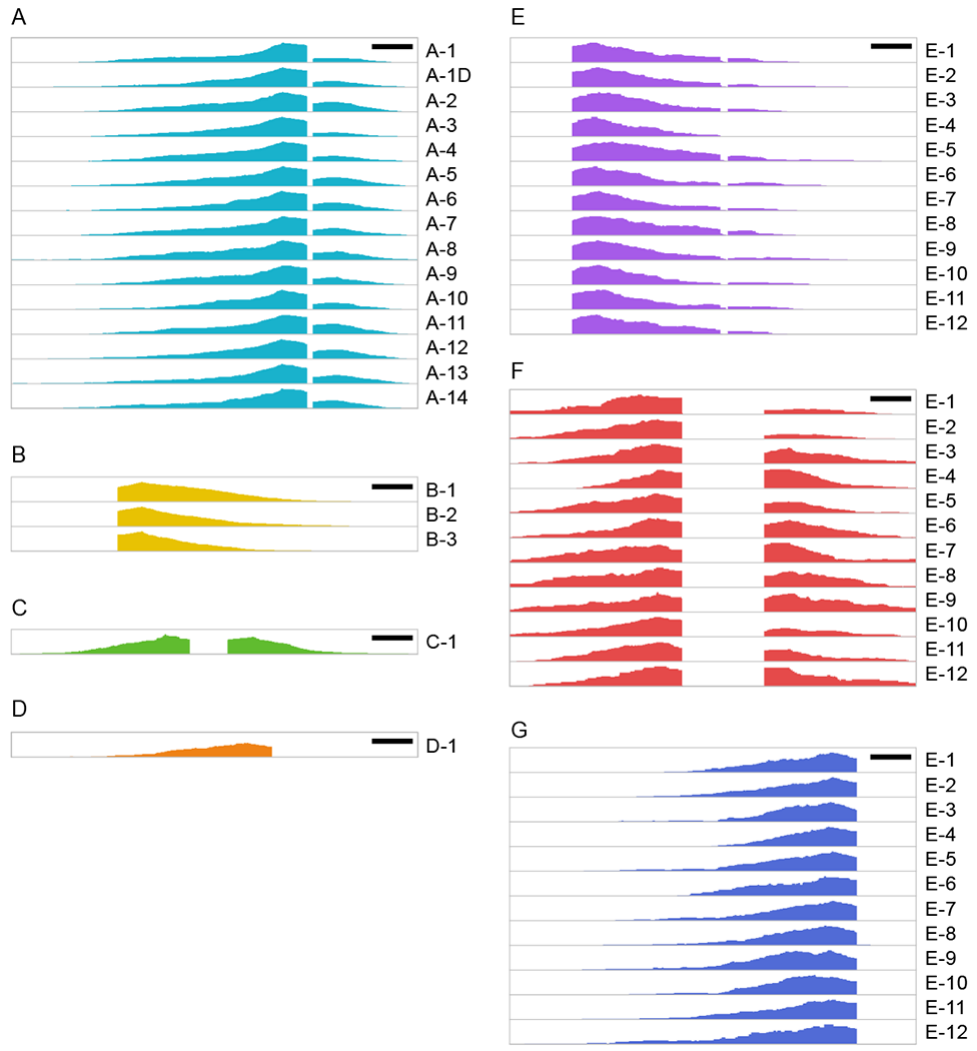
IGV read pileup of identified integration sites for each cell subline. Scale bar represents 50bp and read pileups are colored by scaffold. Loci are listed from left to right as they appear on the pileup. **(A)** Integration loci 1a & 1b on Scaffold T (Chromosome 1) for cells from lineage A. **(B)** Integration locus 2 on Scaffold U (Chromosome 2) for cells from lineage B. **(C)** Integration locus 3 on Scaffold V (Chromosome 6) for cell line from lineage C on the left and unidentified integration locus on the right. **(D)** Integration locus 4 on Scaffold W (Chromosome 5) for cell line from lineage D. **(E)** Integration locus 5 on Scaffold X (Chromosome 3) for cells from lineage E. **(F)** Integration loci 6a & 6b on Scaffold Y (Chromosome 4) for cells from lineage E. **(G)** Integration locus 7 on Scaffold Z (Chromosome 4) for cells from lineage E.

Each integration site was visualized in IGV (Figure 4A–G). The vector-genome junctions are the same at the base by base level for all cell lines from the same lineage, with the read pileup depth decreasing as the distance from the integration site increases.

Only one side of the vector insertion was called by the algorithm for cell line C-1, but on the IGV pileup, both ends are visible, less than 50bp apart (Figure 4C). Upon further investigation, it was determined that the vector side of the split reads at this locus resides in the terminator element that is duplicated in the vector (Figure 1B). Since the vector part of these reads maps identically to two different locations on the vector, BWA was not able to assign an alignment, and these reads were only mapped to the genome. This however does not affect the capacity of the algorithm to verify clonality by integration site analysis; such a site would not map properly in any of the clones. Additionally, not every integration site needs to be identified to show clonality, as the likelihood that two independently derived cell lines would have even a single shared integration site is low. A longer sequencing read length may help avoid these types of non-unique mapping events in the future.

The algorithm did not find integration locus 6b in cell line E-1 (Figure 3C). This integration locus is present in this cell line, as can be seen in Figure 4F, on the right side of the read pileup. The read depth at the integration junction was below the set threshold of five reads, and so the algorithm did not qualify this location as a true integration site. Increased read depth for this sample would have given more confidence in this integration locus.

In this study, we used our method to verify that each subclone had originated from its clonal parent. This workflow can also be used to test clonality or detect non-clonal cells by sequencing a large number of subclones; the presence of subclones with non-consensus integration site(s) would suggest possible non-clonality.

## CONCLUSIONS

The method presented here allows for rapid verification of clonality or common lineage between different cell lines. Use of sequence capture increases the relevant information extracted from sequencing, and the bioinformatic analysis is rapid for processing the low number of reads required from each sample. Additionally, through the use of capture probes targeting common regions of the host vector, future clones can easily be added to the analysis without the need for new probe design. In general, a longer sequencing read length and an increased library size for each sample may have improved this dataset and would be helpful for detecting rarer integration events. The library size may be increased either through reducing the number of samples pooled before capture, or by increasing the input DNA from each sample to reduce PCR duplicates from library preparation. Despite some of these issues in sequencing, the pipeline was robust in its ability to distinguish cell lines of different lineages based on their integration sites. This method is thus a valuable, accessible tool to address clonality verification.

## Supplementary Material

Supplementary Information

## References

[1] Bandyopadhyay AA, Khetan A, Malmberg LH, Zhou W, Hu WS. Advancement in bioprocess technology: parallels between microbial natural products and cell culture biologics. J Ind Microbiol Biotechnol. 2017;44(4-5):785–797. 28185098 10.1007/s10295-017-1913-4

[2] FDA. Points to consider in the manufacture and testing of monoclonal antibody products for human use (1997). U.S. Food and Drug Administration Center for Biologics Evaluation and Research. J Immunother. 1997;20(3):214–243. 9181460 10.1097/00002371-199705000-00007

[3] O'Brien SA, Lee K, Fu HY, Single Copy Transgene Integration in a Transcriptionally Active Site for Recombinant Protein Synthesis. Biotechnol J. 2018;13(10):e1800226. 30024101 10.1002/biot.201800226 PMC7058118

[4] Bandyopadhyay AA, O'Brien SA, Zhao L, Fu HY, Vishwanathan N, Hu WS. Recurring genomic structural variation leads to clonal instability and loss of productivity. Biotechnol Bioeng. 2019; 116(1):41–53. 30144379 10.1002/bit.26823 PMC7058117

[5] Ochman H, Gerber AS, Hartl DL. Genetic applications of an inverse polymerase chain reaction. Genetics. 1988;120(3):621–623. 2852134 10.1093/genetics/120.3.621 PMC1203539

[6] Potter CJ, Luo L. Splinkerette PCR for mapping transposable elements in Drosophila. PLoS One. 2010;5(4):e10168. 20405015 10.1371/journal.pone.0010168 PMC2854151

[7] Schmidt M, Schwarzwaelder K, Bartholomae C, High-resolution insertion-site analysis by linear amplification-mediated PCR (LAM-PCR). Nat Methods. 2007;4(12):1051–1057. 18049469 10.1038/nmeth1103

[8] de Vree PJ, de Wit E, Yilmaz M, Targeted sequencing by proximity ligation for comprehensive variant detection and local haplotyping. Nat Biotechnol. 2014;32(10):1019–1025. 25129690 10.1038/nbt.2959

[9] Arens A, Appelt JU, Bartholomae CC, Bioinformatic clonality analysis of next-generation sequencing-derived viral vector integration sites. Hum Gene Ther Methods. 2012;23(2): 111–118. 22559057 10.1089/hgtb.2011.219 PMC4015219

[10] Giordano FA, Appelt JU, Link B, High-throughput monitoring of integration site clonality in preclinical and clinical gene therapy studies. Mol Ther Methods Clin Dev. 2015;2:14061. 26052530 10.1038/mtm.2014.61 PMC4449016

[11] Aeschlimann SH, Graf C, Mayilo D, Enhanced CHO Clone Screening: Application of Targeted Locus Amplification and Next-Generation Sequencing Technologies for Cell Line Development. Biotechnol J. 2019;14(7):e1800371. 30793505 10.1002/biot.201800371

[12] Ho SC, Bardor M, Feng H, IRES-mediated Tricistronic vectors for enhancing generation of high monoclonal antibody expressing CHO cell lines. J Biotechnol. 2012; 157(1):130–139. 22024589 10.1016/j.jbiotec.2011.09.023

[13] Yusufi FNK, Lakshmanan M, Ho YS, Mammalian Systems Biotechnology Reveals Global Cellular Adaptations in a Recombinant CHO Cell Line. Cell Syst. 2017;4(5):530–542 e536. 28544881 10.1016/j.cels.2017.04.009

[14] Bolger AM, Lohse M, Usadel B. Trimmomatic: a flexible trimmer for Illumina sequence data. Bioinformatics. 2014;30(15):2114–2120. 24695404 10.1093/bioinformatics/btu170 PMC4103590

[15] Rupp O, Macdonald ML, Li S, A reference genome of the Chinese hamster based on a hybrid assembly strategy. Biotechnology and Bioengineering. 2018;115(8):2087–2100. 29704459 10.1002/bit.26722 PMC6045439

[16] Li H Aligning sequence reads, clone sequences and assembly contigs with BWA-MEM. arXiv preprintarXiv:13033997. 2013.

[17] Li H, Handsaker B, Wysoker A, The Sequence Alignment/Map format and SAMtools. Bioinformatics. 2009;25(16):2078–2079. 19505943 10.1093/bioinformatics/btp352 PMC2723002

[18] Thorvaldsdottir H, Robinson JT, Mesirov JP. Integrative Genomics Viewer (IGV): high-performance genomics data visualization and exploration. Brief Bioinform. 2013;14(2):178–192. 22517427 10.1093/bib/bbs017 PMC3603213

