# Supplementary material for: Multiplexed clonality verification of cell lines for protein biologic production
Source: Biotechnol Prog. Author manuscript; Available in PMC 2021 Jul 1. (PMC7803388; doi:10.1002/btpr.2978)
Supplement: Supplementary Information [file NIHMS1652551-supplement-Supplementary_Information.pdf]

SUPPLEMENTARY TABLES

Table S1. Read mapping statistics for sequence capture data from SH-87. Each end of the paired end read is reported separately as R1 and R2.

	**R1**	**R2**
**Number of Unique Reads (Library Size)**	2,391,462	2,391,462
**Reads mapping entirely to Vector**	18.4%	18.4%
**Reads mapping entirely to Genome**	80.9%	80.0%
**Split reads between vector and Genome**	0.192%	0.175%
**Low quality split reads**	0.105%	0.099%
**Unmapped reads**	0.4%	1.4%

Table S2. Integration sites confirmed by algorithm for cell line SH-87. CHO-K1 Genome integration sites obtained from Yusufi, et al. 2016.[13]

**Site**	**CHO-K1 Genome Scaffold**	**Scaffold Position**	**CriGri-PICR Genome Scaffold**	**Scaffold Position**	**Vector Position**
A	NW_003613840.1	743320	NW_020822636.1	2762822	9
B	NW_003613840.1	747517	NW_020822636.1	2767055	505
D	NW_003613840.1	747631	NW_020822636.1	2767169	654
E	NW_003614673.1	136752	NW_020822636.1	898236	4466
C	NW_003614673.1	141082	NW_020822636.1	902565	1226
F	NW_003616992.1	80949	NW_020822636.1	177576	73

Table S3. Description of integration sites called by the algorithm for clonality analysis.

Site #	Cell Lines	Genome Location	Vector Junction
1a	All lineage A cell lines	Chromosome 1, exon, protein-coding	Between 2nd poly A and bacterial resistance
1b	All lineage A cell lines	Chromosome 1, exon, protein-coding	Between 1st poly A and 2nd promoter
1c	A-2	Chromosome 1, exon, predicted long non-coding RNA	Between 1st poly A and 2nd promoter
2	All lineage B cell lines	Chromosome 2, intron, protein-coding	Puromycin-N-acetyltransferase gene
3	C-1	Chromosome 6, intergenic region	Bacterial resistance on 3’ end of linearized vector
4	D-1	Chromosome 5, intron, protein-coding	Between 2nd poly A and bacterial resistance
5	All lineage E cells lines	Chromosome 3, exon, protein-coding	Bacterial resistance on 5’ end of linearized vector
6a	All lineage E cells lines	Chromosome 4, intergenic region	2nd poly A
6b	All lineage E cell lines except E-1	Chromosome 4, intergenic region	Bacterial resistance on 5’ end of linearized vector
6c	E-2, E-10	Chromosome 4, intergenic region	1st promoter
7	All lineage E cells lines	Chromosome 4, exon, protein-coding	Bacterial resistance on 5’ end of linearized vector

SUPPLEMENTARY FIGURES

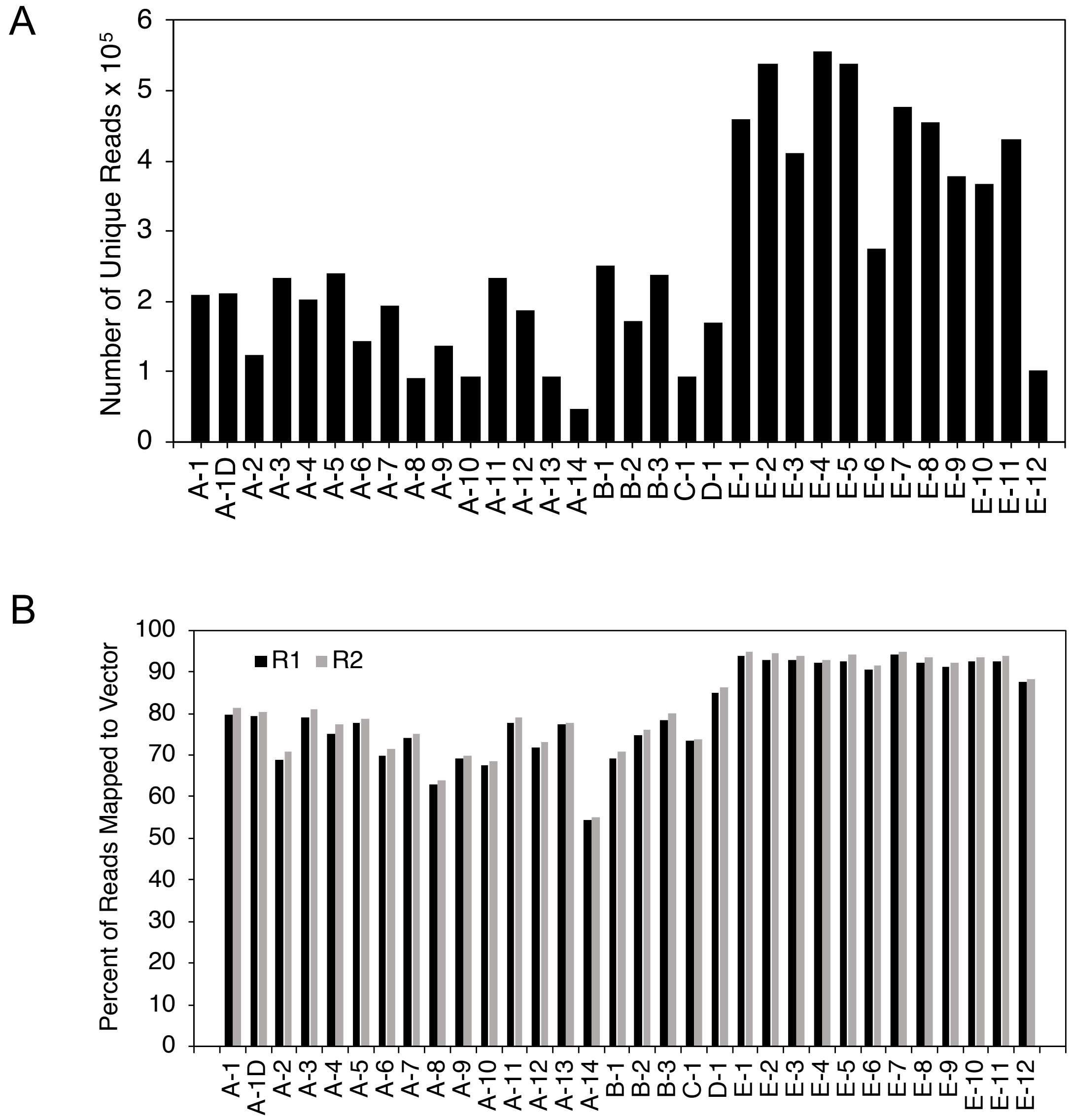

Figure S1. Sequencing statistics for cell lines used for clonality analysis. **(A)** Number of unique reads for each cell line after removal of PCR duplicates. **(B)** Percent of reads mapped to the vector for each cell line. Each end of the paired end read is reported separately as R1 and R2.
